# *Streptococcus suis*–Associated Meningitis, Bali, Indonesia, 2014–2017

**DOI:** 10.3201/eid2512.181709

**Published:** 2019-12

**Authors:** Ni Made Susilawathi, Ni Made Adi Tarini, Ni Nengah Dwi Fatmawati, Putu I.B. Mayura, Anak Agung Ayu Suryapraba, Made Subrata, Anak Agung Raka Sudewi, Gusti Ngurah Mahardika

**Affiliations:** Udayana University, Denpasar, Bali, Indonesia

**Keywords:** Streptococcus suis, human, meningitis/encephalitis, Bali, Indonesia, bacteria, pork, swine, zoonoses

## Abstract

*Streptococcus suis* is an emerging agent of zoonotic bacterial meningitis in Asia. We describe the epidemiology of *S. suis* cases and clinical signs and microbiological findings in persons with meningitis in Bali, Indonesia, using patient data and bacterial cultures of cerebrospinal fluid collected during 2014–2017. We conducted microbiological assays using the fully automatic VITEK 2 COMPACT system. We amplified and sequenced gene fragments of glutamate dehydrogenase and recombination/repair protein and conducted PCR serotyping to confirm some serotypes. Of 71 cases, 44 were confirmed as *S. suis*; 29 isolates were serotype 2. The average patient age was 48.1 years, and 89% of patients were male. Seventy-seven percent of patients with confirmed cases recovered without complications; 11% recovered with septic shock, 7% with deafness, and 2% with deafness and arthritis. The case-fatality rate was 11%. Awareness of *S. suis* infection risk must be increased in health promotion activities in Bali.

## Abstract 2

Health promotion activities should focus on increasing awareness of risk for infection.

Community-acquired bacterial meningitis is a serious infectious disease with high rates of illness and death worldwide, even in the era of effective antimicrobial drugs (*1*). The disease is classified as a neurologic emergency; thus, immediate diagnosis and accurate treatment are vital to save the patient’s life (*2*). Gram-positive, coccus-shaped *Streptococcus suis* (*3*) is the most common causative agent of zoonotic bacterial meningitis; pigs are the primary source of infection. *S. suis* is an important pathogen in community-acquired bacterial meningitis (*2*,*4*,*5*).

Human *S. suis* infections are mostly associated with pig husbandry and eating pork-derived products. Since 2010, the number of reported *S. suis* infections in humans has increased substantially; most cases have originated in Southeast Asia, where the density of pigs is high (*6*). Moreover, >1,600 *S. suis* infections have been reported in 30 countries worldwide (*7*). Previously considered to be sporadic, *S. suis* meningitis can cause epidemics, as occurred in Thailand, Vietnam, and China (*3*). The presence of this bacterium is likely to be inevitable in areas with dense pig populations, including the province of Bali in Indonesia. We describe data on the epidemiology, clinical signs, and microbiology of *S. suis* from meningitis cases in Bali.

## Materials and Methods

### Data Collection

We obtained medical records of persons who had suspected bacterial meningitis during 2014–2017 from the Sanglah Provincial Referral Hospital (SPRH; Denpasar, Bali, Indonesia). SPRH is a 760-bed national referral hospital for eastern Indonesia with >600,000 annual visits.

Cerebrospinal fluid (CSF) was collected from each patient at admission. Recorded data included patient demographics and clinical signs indicating bacterial meningitis, such as altered mental status, fever, headache, and neck stiffness (*8*). Other data were CSF laboratory test results, therapy history, and outcomes.

### Laboratory Investigation

We cultured CSF samples from patients with suspected meningitis on a 5% defibrinated sheep blood agar plate (DSBAP) and incubated in 5% CO_2_ at 37°C for 18–24 h (*9*). We isolated colonies for identification and drug susceptibility testing using fully automatic VITEK 2 COMPACT system (bioMérieux, https://www.biomerieux.com) based on Clinical and Laboratory Standards Institute guidelines (*10*). Upon positive detection, we grew selected colonies in tryptic soy broth, incubated at 37°C for 18–24 h, and preserved at –80°C in 50% glycerol. We cultured 44 glycerol stock isolates of *S. suis* on DSBAP and incubated in 5% CO_2_ at 37°C for 18–24 h for further study. We reconfirmed the bacterial identity using VITEK 2 COMPACT.

### PCR and Sequencing

We suspended 6–8 colonies grown on DSBAP in 200 μL of phosphate buffered saline (pH 7.3) and then isolated bacterial DNA using a Roche High Pure PCR Template Isolation Kit (Roche Life Science, https://www.roche.com). DNA was eluted with 50 μL of elution buffer. We confirmed all isolates by PCR using glutamate dehydrogenase (GDH) and recombination/repair protein (recN) primer sets, as described previously (*11*,*12*). We commercially sequenced selected PCR products in 1stBase (Selangor, Malaysia), aligned them using MEGA 6.0 (https://www.megasoftware.net), and subjected them to BLAST search (https://blast.ncbi.nlm.nih.gov/Blast.cgi). We inferred phylogenetic reconstruction of GDH sequence using the unweighted pair group method with arithmetic mean (*13*). We downloaded the GDH or parts of complete genomes of *S. suis* from GenBank for reference and included 1 sequence of *S. pneumoniae* in the phylogenetic analysis. We conducted PCR serotyping to confirm serotype 2 and 1/2, as well as 1 and 14, using published primer sets (*14*). Further differentiation of serotype 2 to serotype 1/2 was based on BLAST of recN.

### Ethics Approval

The Research Ethics Committee of the Faculty of Medicine, Udayana University (Denpasar, Bali, Indonesia), approved this study (no. 691/UN.14.2/KEP/2017, dated April 7, 2017). In accordance with the standard operation procedure of the SPRH, CSF was collected after informed consent.

## Results

**Figure 1:**
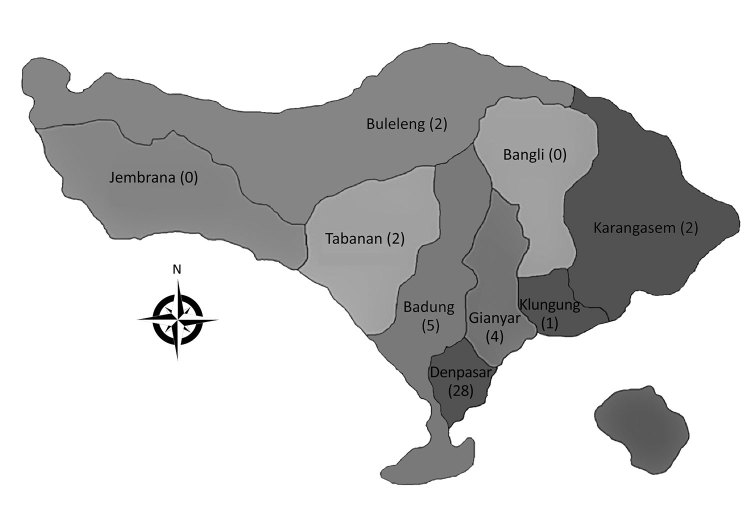
Geographic origin of patients in each regency/municipality confirmed to have *Streptococcus suis* meningitis in Sanglah Provincial Referral Hospital, Denpasar, Bali, Indonesia, 2014–2017. Numbers of patients are shown in parentheses.

Of 71 acute bacterial meningitis cases, *S. suis* was confirmed in CSF culture of 44 patients (Table 1). The median time from illness onset to hospital admission was 2 days (range 1–14 days). Thirty-nine (89%) patients were male; the average patient age + SD was 48.1 + 11.5 (range 28–77 years). The most common 3 municipalities/regencies of origin of patients were Denpasar (28 [64%] confirmed cases), Badung (5 [11%]), and Gianyar (4 [9%]) (Figure 1). Patient occupations were private sector employees (57%), unemployed (14%), farmers (11%), entrepreneurs (11%), and government employees (7%).

**Table 1:** Demographic data for patients confirmed to have *Streptococcus suis* meningitis, Sanglah Provincial Referral Hospital, Bali, Indonesia, 2014–2017*

Variable	Value
Onset of illness before hospital admission, median d (range)	2 (1–14)
Sex	
M	39 (88.6)
F	5 (11.4)
Age, y, mean ± SD (range)	48.1 ± 11.5 (28–77)
Origin	
Denpasar	28 (63.6)
Badung	5 (11.4)
Gianyar	4 (9.1)
Buleleng	2 (4.5)
Karangasem	2 (4.5)
Tabanan	2 (4.5)
Klungkung	1 (2.3)
Jembrana	0
Bangli	0
Employment	
Private sector employee	25 (56.8)
Unemployed	6 (13.6)
Farmer	5 (11.4)
Entrepreneur	5 (11.4)
Government employee	3 (6.8)

*Values are no. (%) patients unless otherwise indicated.

The 4 most frequent clinical signs in patients with acute *S. suis* meningitis were fever (91%), neck stiffness (86%), altered mental status (86%), and headache (82%) (Table 2). Septic shock was documented in 5 (11%) cases and sensorineural hearing loss in 4 (9%); seizure, ataxia, and hemiparesis were each recorded in 3 cases (7%) and arthritis in 2 (5%).

**Table 2:** Clinical signs and outcomes of patients with confirmed *Streptococcus suis* meningitis, Sanglah Provincial Referral Hospital, Bali, Indonesia, 2014–2017

Variable	No. (%) patients
Sign	
Fever	40 (90.9)
Neck stiffness	38 (86.4)
Altered mental status	38 (86.4)
Headache	36 (81.8)
Nausea/vomiting	13 (29.5)
Septic shock	5 (11.4)
Sensorineural hearing loss	4 (9.1)
Seizure	3 (6.8)
Ataxia	3 (6.8)
Hemiparesis	3 (6.8)
Septic arthritis	2 (4.5)
Definitive diagnosis *S. suis* acute bacterial meningitis with:	
No complications	34 (77.3)
Septic shock	5 (11.4)
Deafness	3 (6.8)
Signs of relapse*	2 (4.5)
Deafness and arthritis	1 (2.3)
Outcome	
Full recovery	32 (72.7)
Moderate disability	7 (15.9)
Death	5 (11.4)

*Relapsed meningitis: not recovered after 14 d treatment, but responded well after prolonged (3 weeks) ceftriaxone treatment.

All patients were treated intravenously with 2 g of ceftriaxone every 12 hours for 14 days and 10 mg of dexamethasone every 6 hours for 4 days. In 2 patients, meningitis relapsed after 14 days of ceftriaxone treatment, but they recovered after 3 additional weeks of ceftriaxone therapy. The case-fatality rate (CFR) was 11%; moderate disabilities occurred in 16% of survivors in the form of sensorineural deafness (4 patients) and hemiparesis (3 patients).

Complete blood counts showed leukocytosis (mean + SD 24.4 + 10.5 × 10^3^ cells/μL) (Table 3). The neutrophil differential count was 88.4% + 9.8%, and the lymphocyte count was 4.9% + 4.7%. The mean platelet count was 196.4 + 100.2 × 10^3^ cells/μL. CSF analysis showed pleocytosis (median 799 cells/μL; range 92–8,510 cells/μL); CSF neutrophil count was 60%, and lymphocyte count was 40%. Glucose levels were low (median 5 mg/dL; range 0–78 mg/dL); the CSF/blood glucose ratio was 0.4; and protein levels were increased (median 198 mg/dL; range 64–855 mg/dL). CSF culture was positive for *S. suis* and sensitive to ceftriaxone, benzyl-penicillin, ampicillin, levofloxacin, erythromycin, vancomycin, and linezolid (data not shown).

**Table 3:** Laboratory findings in *Streptococcus suis* meningitis patients, Sanglah Provincial Referral Hospital, Bali, Indonesia, 2014–2017

Parameters	Finding	Reference values
Blood		
Leukocytes, × 1,000/μL, mean ± SD	24.4 ± 10.5	4.1–11.0
Neutrophils, no. (%)	88.4 (9.8)	47–80
Lymphocytes, no. (%)	4.9 (4.7)	13–40
Platelet count, × 1,000/μL, no. (%)	196.4 (100.2)	140–440
Cerebrospinal fluid, median (range)		
Cell count, cells/μL	799 (92–8,510)	0–5
Glucose, mg/dL	5 (1–78)	60–80
Blood/glucose ratio	0.4 (0.1–74)	>0.66
Protein, mg/dL	198 (64–855)	<45

**Figure 2:**
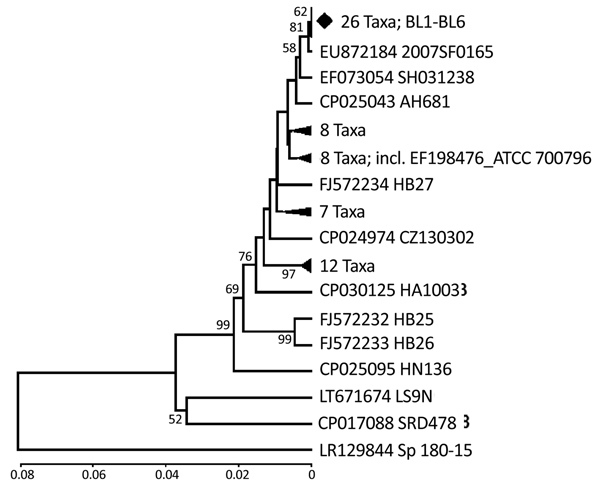
Phylogenetic relationships of the glutamate dehydrogenase gene fragment of *Streptococcus suis* isolated from humans in Denpasar, Bali, Indonesia (BL1-BL6 taxa), with sequences data of *S. suis* available in GenBank. The phylogeny was inferred using unweighted pair group method with arithmetic mean (*13*). The GenBank accession number and strain name are written as taxon name. To minimize crowding, some tree branches were condensed. The number of taxa in each condensed branch is indicated. The location of standard American Type Culture Collection isolate (GenBank accession no. EF198476) is shown. Respective gene sequence of full-genome data of *S. pneumoniae* (accession no. LR129844) was co-analyzed as outgroup. The percentage of replicate trees in which the associated taxa clustered in the bootstrap test (1,000 replicates) are shown next to the branches (*16*). Bootstrap values of <50% are not shown. The genetic distances were computed using the Kimura 2-parameter method (*17*). Phylogenetic analyses were conducted in MEGA6 (*18*). Scale bar indicates nucleotide substitutions per site.

PCR results for GDH and recN of all samples produced specific single bands of expected sizes (data not shown). Five GDH and 3 recN PCR products were sequenced. The sequences of GDH and recN generated in this study are available in GeneBank (accession nos. MK161045–54). All GDH and recN sequences of *S. suis* generated in our study were identical. BLAST analysis of the GDH sequence, using blastn (*15*), demonstrated that the sequence had query cover of 100% and an identity score of 94%–100% with the complete *S. suis* genome, GDH complete or partial cDNA sequences (CDS). *S. pneumoniae* and *S. marmotae* had a 99%–100% query cover and an identity score of 86%. For recN, the sequence from the isolates had query cover of 100% and identity of 95%–99% with the *S. suis* complete genome and *S. suis* recN partial CDS. The next closest query cover of 54% with identity score of 83% was with the recN CDS of *S. parasuis*. Phylogenetic analysis of GDH (Figure 2) showed that the isolates were identical with 20 GDH and part of complete genome sequences of *S. suis*.

PCR serotyping showed that 29 (66%) of the 44 isolates were positive in PCR using primer pair for serotype 2 or 1/2, which amplifies *cps2I* gene, whereas none were positive using primer pair for serotypes 1 and 14 detecting *cps1I*, as previously published (*14*). The sequence of *cps2I* of our isolates are available in GenBank (accession nos. MN395406–34). The readable length of sequences was 284 bp. The sequences were identical to *S. suis cps2I* gene of the reference sequence (GenBank accession no. KC537364) (*14*). BLAST analysis showed the recN of our isolates were distancing 3.7% to the strain 2651 (GenBank accession no. AB724091), which was annotated as serotype 1/2 (*19*).

## Discussion

Our study confirms that *S. suis* is present and infects human in Bali. This finding should alert other provinces in Indonesia. The bacterium has been isolated previously in other provinces (*20*,*21*), but the cases of human *S. suis* meningitis we report extend the known range of *S. suis* in Indonesia. Pigs are raised in many provinces in Indonesia, and densities differ. In 13 provinces, pig populations were >100,000 head in 2017 (https://www.bps.go.id). The presence of *S. suis* in other provinces needs to be confirmed. Pigs or pig products are thought to be the main source of human infection (*6*) because evidence on the role of other species is unavailable. The awareness will be invaluable in avoiding human suffering and death because medical services will be fully informed and aware of the risk posed by *S. suis* and thus better equipped to save lives.

We based this study on medical records of persons with suspected bacterial meningitis during 2014–2017 at SPRH. All patients with suspected meningitis in the province are referred to this hospital for a definitive diagnosis. Although the presence of *S. suis* has been confirmed only since 2014, suspected bacterial meningitis had been suspected before then and diagnosed as *S. viridans* group. The installment of VITEK 2 COMPACT testing confirmed *S. suis* in 2014. Although cases from many districts in Bali might have been underdiagnosed, we believe that the number of confirmed cases in this report represents most human cases in the province.

Handling pigs or pork products seems to be the major risk factors for human transmission of *S. suis* (*22*). Pork products can originate from slaughterhouses, as has been described in Vietnam (*23*), or from backyard slaughter of dead or sick pigs, as reported in China (*24*). The risk also seems to increase when raw pork products are eaten. Furthermore, eating raw or medium-cooked pork-derived food containing blood, tonsil, tongue, intestine, and uterus has been indicated as an important risk factor for *S. suis* meningitis (*25*,*26*). A history of ingesting raw pork, pig’s blood, or both was found in most cases in Thailand (*27*,*28*).

Eating raw meat with fresh blood from sick or subclinically infected pigs might be a major risk factor for *S. suis* transmission in humans in Bali, Indonesia. Most (88%) confirmed *S. suis* meningitis patients in our study were men. This finding was similar to that of *S. suis* infection in Thailand (*28*). The average age and the proportion of men is consistent with the results of a systematic review of studies published during 1980–2015 (*2*). The link of traditional pork consumption and pig handling to the risk for contracting *S. suis* needs to be elucidated further in Bali.

*S. suis* was predominant as the causal agent of acute bacterial meningitis in our study. Our finding shows it was confirmed in 44 (62%) of 71 acute bacterial meningitis cases. The percentage might have been higher because the *S. suis*–negative patients received antimicrobial therapy before sampling. Human infection with this bacterium needs immediate interventions. Recent data from SPRH showed 20 confirmed cases in 2018 and 13 as of July 2019.

Clinical signs of *S. suis* meningitis recorded in this study resemble those of general bacterial meningitis (*2*,*4*,*6*,*29*). All cases were of acute infection. The median time from illness onset to hospital admission in our study was 2 days (range 1–14 days). The 4 most frequent clinical signs were fever or history of fever, neck stiffness, altered mental status, and headache; these signs correspond to the 3 most frequent globally reported symptoms of meningitis: fever, headache, and neck stiffness (*2*,*30*).

Initially, some patients did not demonstrate overt neurologic symptoms and thus were admitted under nonneurologic diagnoses. One patient was admitted to the Ear, Nose and Throat Department for sensorineural bilateral deafness, and another was admitted as having an ischemic stroke. Another patient was admitted with suspected dengue fever, which later developed into clinical meningitis. Such misadmission is understandable and may be more widespread because infection with *S. suis* has been reported to cause other syndromes, such as arthritis, endocarditis, peritonitis, and endophthalmitis (*29*–*31*).

If we grade outcomes according to the Glasgow Outcome Scale (*32*), 73% of the patients in our study had favorable outcomes. All 44 patients were intravenously treated for bacterial meningitis with 2 g of ceftriaxone every 12 hours for 14 days and 10 mg of dexamethasone every 6 hours for 4 days, in accordance with SPRH protocol. Ceftriaxone is a third-generation cephalosporin, which is recommended as the drug of choice for bacterial meningitis (*6*,*8*).

The CFR in our study was 11%; death was caused by septic shock, which has been attributed to *S. suis* infection (*2*,*33*). The CFR here is slightly higher than the globally reported CFR of ≈3% (*2*). The reported CFR for *S. suis* meningitis is lower than for other bacterial meningitis, such as pneumococcal (20%) and *Listeria monocytogenes* (36%) meningitis (*2*). The relatively high CFR seems to be related to the late admission of some patients in our study. A high CFR has also been reported in Thailand (*34*).

Four (9%) patients reported hearing loss in our study. This percentage is lower than that from previous findings. In a systematic review and meta-analysis to summarize global estimates of the epidemiology, clinical characteristics, and outcomes of *S. suis* infection, hearing loss was reported in ≈40%–50% of cases and vestibular dysfunction in >20% (*2*,*29*). This discrepancy might be due to early administration of antimicrobial drugss, so *S. suis* was uncultivable. Also, we excluded unconfirmed cases from our study.

Laboratory findings in the CSF were leukocytosis (predominantly neutrophil), low glucose levels, and increased protein content. These findings resemble typical bacterial meningitis (*1*,*8*).

Of all *S suis* serotypes, serotype 2 is recognized as the most common pig and human pathogen (*23*,*35*). However, other serotypes should not be ignored, as evidenced by serotype 5 in Japan (*36*), serotype 9 in Thailand (*37*), serotype 16 in Vietnam (*38*), serotype 21 in Argentina (*39*), serotypes 24 (*40*) and 31 (*41*) in Thailand, and many more. PCR serotyping indicated that 29 of 44 isolates were positive in PCR using a primer set to detect serotypes 2 and 1/2 (*14*) but not serotypes 1 and 14. We focused on serotypes 2 and 1/2 because *S. suis* serotype 2 is the most common cause of human cases (*42*); serotypes 1, 4, 14, and 16 infection can lead to severe illness, but fewer cases are reported than for serotype 2 (*38*). We confirmed those PCR-positive isolates in our study to be serotype 2 or 1/2. The readable sequences were identical to *S. suis cps2I* gene of the reference sequence (GenBank accession no. KC537364) (*14*). Although the existing PCR serotyping is unable to differentiate between serotype 2 and 1/2 (*14*), the nucleotide sequences of recN of our isolates are distancing 3.7% to the 2651 strain (GenBank accession no. AB724091), which was annotated as serotype 1/2 (*19*). Therefore, we proposed those PCR-positive isolates were serotype 2. Samples should be sent to a reference laboratory to be tested using a panel of standard antiserum (*6*), and the complete primer sets for PCR serotyping (*14*) serotypes of all isolates should be made available. The knowledge gained will convey important epidemiologic picture for human prevention.

We confirmed *S. suis* in this study after applying a standard method with fully automatic equipment. Performing PCR and sequencing of GDH and recN further confirmed the species identification. Both gene fragments are proposed as an appropriate PCR system for the reclassification of *S. suis* (*11*) or as a specific PCR system for *S. suis* (*12*).

BLAST search of the GDH sequences showed high coverage and identity with the *S. suis* complete genome and GDH partial CDS available in the database. The closest identity score of 86% was to *S. pneumoniae* and *S. marmotae*. Phylogenetic analysis (Figure 2) also confirmed that our isolates are *S. suis.* The recN had high sequence coverage and high identity to the *S. suis* database, too., The closest sequence data of *S. parasuis* have an identity score of 83% to the recN of *S. suis*. Sequencing of PCR products to confirm detected genetic sequences should limit or reduce misidentification. We did not sequence all PCR products because sequencing was conducted only to determine the specificity of the PCR. We propose implementation of GDH and recN as diagnostic tools in elucidating the distribution of *S. suis* in Indonesia.

Misidentification of *S. suis* is common. This bacterium is frequently misidentified as *S. viridans* (*43*) and has also been misidentified as *S. bovis*, *S. pneumoniae*, *S. faecalis*, and *S. acidominimus* (*29*,*44*). Misidentification of *S. suis* also has been reported in Canada, which raises suspicion that human *S. suis* infections might be underdiagnosed in North America (*45*). We found 1 case of suspected *S. mitis* infection using the VITEK 2 COMPACT system. However, PCR and sequencing confirmed this to be *S. suis*.

Published reports of animal cases and isolation of *S. suis* from animals in Bali are not available. Isolation of *S. suis* from tonsil samples has been reported from Papua, Indonesia (*21*). Another group in Udayana University is working to isolate and detect *S. suis* from sick pigs in Bali, further suggesting that *S. suis* is present in the island (K. Besung, Udayana University, pers. comm., 2018 Oct 1)*.* As indicated elsewhere that pig and pork products are the primary sources of human infection (*2*,*4*,*5*), so is the source of *S. suis* in humans in our study most likely to be pigs and pork products.

In conclusion, we confirmed *S. suis* meningitis in humans in Bali, Indonesia. Of 44 cases, 29 human isolates were serotype 2. Because human infections are mostly associated with pig husbandry and eating pork-derived products, the distribution of *S. suis* in the country needs to be fully elucidated. The risk factor of eating raw pork and pig blood in traditional delicacies seems to be valid, although this point requires further investigation. Our study contributes to enhancing knowledge of *S. suis* distribution and risk factors in Bali. By increasing awareness of *S. suis* infection, medical services will be better prepared to alleviate human suffering and death from *S. suis* meningitis.
